# Application of Instrumented Paddles in Measuring On-Water Kinetics of Front and Back Paddlers in K2 Sprint Kayaking Crews of Various Ability Levels

**DOI:** 10.3390/s20216317

**Published:** 2020-11-05

**Authors:** Pui Wah Kong, Cheryl Sihui Tay, Jing Wen Pan

**Affiliations:** Physical Education and Sports Science Academic Group, National Institute of Education, Nanyang Technological University, Singapore 637616, Singapore; cheryltsh@gmail.com (C.S.T.); NIE173748@e.ntu.edu.sg (J.W.P.)

**Keywords:** force, power, temporal, stroke, offsets, water sports, crew boat

## Abstract

This study used instrumented paddles to obtain on-water kinetic variables of two-seater (K2) crews during sprint kayaking. A total of 74 male kayakers of various ability levels (national team: 9, recreational club: 38, school team: 27) comprising 39 K2 crews were recruited. Both the front and back paddlers were provided with an instrumented paddle to perform 200-m maximal effort paddling in a reservoir. Force, power, and temporal variables were extracted from the paddle data. Difference among groups were compared using a factorial Analysis of Variance. Results showed that the force, power, and temporal characteristics of the front and back paddlers were similar during maximal effort sprint kayaking. Proficient kayakers produced greater kinetic outputs than less proficient kayakers, while the coordination strategy based on timing differences at key events between the two crew members in a K2 boat was similar across ability levels. These data can be useful for coaches, sport scientists, and athletes in planning and monitoring the training.

## 1. Introduction

Sprint kayaking is an Olympic sport in which athletes compete in one-seater kayak (K1), two-seater (K2), or four-seater (K4) crew boats. To win a race, paddlers are required to perform powerful strokes alternatively on the left and right sides to generate high mean boat velocity over a race distance. In the past few decades, various types of sensors and technologies have been developed to measure the stroke kinetics during on-water kayaking. Earlier prototypes measured paddling forces using strain gauges of which the working principle is that bending moments have a strong linear relationship with the resistance [1,2]. Later on, the FPaddle system was invented incorporating strain gauges, triaxial accelerometers, and wireless data transmission system [3,4]. In addition to measuring force output, the Kayak Power Meter (One Giant Leap, Nelson, New Zealand) is also able to quantify stroke power via strain gauges and gyroscopes embedded onto the carbon fibre paddle shaft [5]. Stemming from optical fibre technology, Niu and colleagues [6] proposed a real-time compression load measurement system for a kayaking paddle using fibre Bragg grating (FBG). This FBG-based instrumented paddle successfully measured handgrip force and blade loading distribution on competitive and recreational kayakers in a 50-m swimming pool [7]. Recently, a e-Kayak system with multichannel digital acquisition system was tailor-made for flatwater sprint kayaking to simultaneously measure forces acting on the paddle and footrest as well as kinematics of the paddler and the boat [8,9].

Nearly all previously studies of on-water kayak performance examined individual performance in a one-seater K1 kayak [3,5,7,9,10]. The literature on crew boats is very limited [11,12,13]. Gomes [11] compared the force profiles of 11 elite kayakers when paddling in one-seater K1 and both front and back seats of a K2 crew boat. It was reported that the kinetic outputs in K2 were lower than that in K1. Tay and Kong [12,13] used video analysis to quantify the timing offsets between the front and back paddlers in eight K2 crews during high intensity paddling. Bonaiuto et al. [8] proposed a possible digital acquisition system to monitor K2 stroke performance of two paddlers but only data of a single female athlete were reported. To the best of authors’ knowledge, there is currently no large-scale investigation into the kinetic profiles of K2 crew members in sprint kayaking. The application on instrumented kayak paddles is a promising way to monitor on-water kayaking stroke performance. Of the few available instrumented paddle systems, Kayak Power Meter is considered the best option because (1) it is commercially available, (2) it measures both stroke force and power, and (3) it allows synchronisation of two or more sets of the system, making it possible to quantify the contribution of individual kayaker as well as the timing offsets between the front and back paddlers. Using the Kayak Power Meter, the present study aimed to (1) compare the kinetic profiles of the front and back paddlers in a large sample of K2 sprint kayaking athletes, and (2) compare the kinetic outputs between kayakers of varied abilities. As the present study pioneers the application of instrumented paddles in on-water crew boat measurement, findings could contribute towards a better understanding of crew boat sprint kayaking and future instrumentation of sports equipment.

## 2. Materials and Methods

### 2.1. Kayak Power Meter

**Figure 1:**
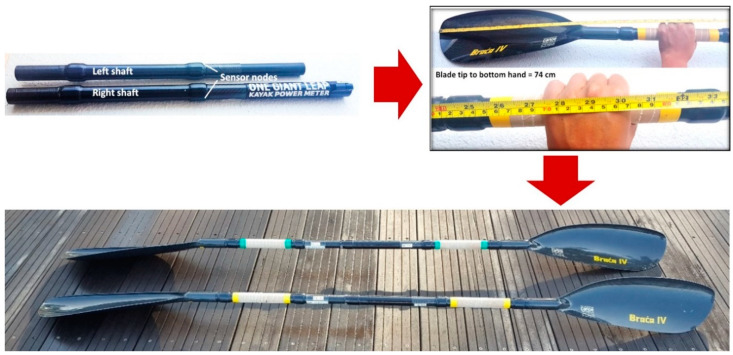
The Kayak Power Meter was designed as an adjustable split-shaft system, with the right shaft being the master control to allow up to 5 cm of paddle length adjustment and preferred blade feather angles. When assembled with paddle blades, the overall mass was 1.010 kg, approximately 12% heavier than a regular paddle of 0.901 kg.

The Kayak Power Meter (One Giant Leap, Nelson, New Zealand), a commercially available product, was adopted in the present study to measure stroke force and power (Figure 1). This system was designed as an adjustable split-shaft system, with the right shaft being the master control allowing up to 5 cm of paddle length adjustment and preferred blade feather angles. The instrumented draft was made of carbon fibre, the same material as a regular shaft. While a regular shaft is hollow, the instrumented shaft is embedded with sensors (strain gauges and gyroscopes) and hence is heavier. The mass of the instrumented paddle shaft was 0.394 kg, which was 38% heavier than a regular shaft (approximately 0.285 kg). When assembled with paddle blades, the overall mass was 1.010 kg, approximately 12% heavier than a regular paddle of 0.901 kg. The validity of the Kayak Power Meter was reported to be within 0.12% to 1.4% of the true value, while the reliability ranged from 0.27% to 0.34% for an applied force of 155.9 N [5]. According to manufacturer specifications, the typical measurement resolutions were 1.0 N for the top hand and 2.5 N for the bottom hand, with a range of 780 N to 2100 N.

Prior to usage, the Kayak Power Meter must be set up using the proprietary Windows Desktop App (version 1.1.9.10). An Advanced and Adaptive Network Technology (ANT+™) Universal Serial Bus (USB) stick was connected to the computer to run the Windows Desktop App. For the same set of the Kayak Power Meter, two athlete-specific settings were customised for each user: blade tip to top hand distance, and blade tip to bottom hand distance (Figure 1). Both measurements were taken to the middle knuckle of each hand. As the hand positions were important for the calculations of force and power, it was recommended that grip positions were marked out with a tape for easy reference.

Data recording may be triggered either through the Windows Desktop App or a paired compatible ANT+™ device operating in bicycle power mode. There were two options for sampling rates. In the normal mode (1 Hz), stroke rate, and power are visible in real-time on the ANT+™ device. These data are recorded in the ANT+™ device memory and can be downloaded using the procedures for the specific device. In the high-speed mode (50 Hz), up to 12-min of stroke force and power data are recorded to the nearest integer and stored within the memory chip of the Kayak Power Meter. This mode must be pre-set in the Windows Desktop App by selecting the recording duration. The recorded high-speed data are transmitted and downloaded wirelessly through the Windows Desktop App. In the present study, the high-speed data mode of 50 Hz was chosen as the shape of the force and power curves over time are of interest.

### 2.2. Participants

This study was approved by the Nanyang Technological University Institutional Review Board (NTU-IRB-2016-03-001). Study procedures were performed in accordance with the Helsinki Declaration on research involving human participants. Participants provided written informed consent prior to any study procedures. For minor participants below the age of 21 years, written parental or guardian informed consent and minor assent were obtained. A convenience sample of 74 (9 from the national team + 38 from recreational clubs + 27 from school teams = 74) male sprint kayakers comprising 39 K2 crews participated in this study (Table 1). The participants comprised a wide range of proficiency levels, and were recruited from school teams, recreational clubs, and the Singapore national team. They were all healthy and injury-free during the study duration. Three national team kayakers (participant 73, 74, and 75) formed three crews: crew 37 (front–back, 73–74), crew 38 (75–74), and crew 39 (73–75) with their preferred seat orders, and hence six crews, in total, were assigned to the national team. The national team kayaker (participant 41) was also paired with a school team kayaker (participant 138) to form a crew in the school team (crew 71).

### 2.3. Experimental Protocols

**Figure 2:**
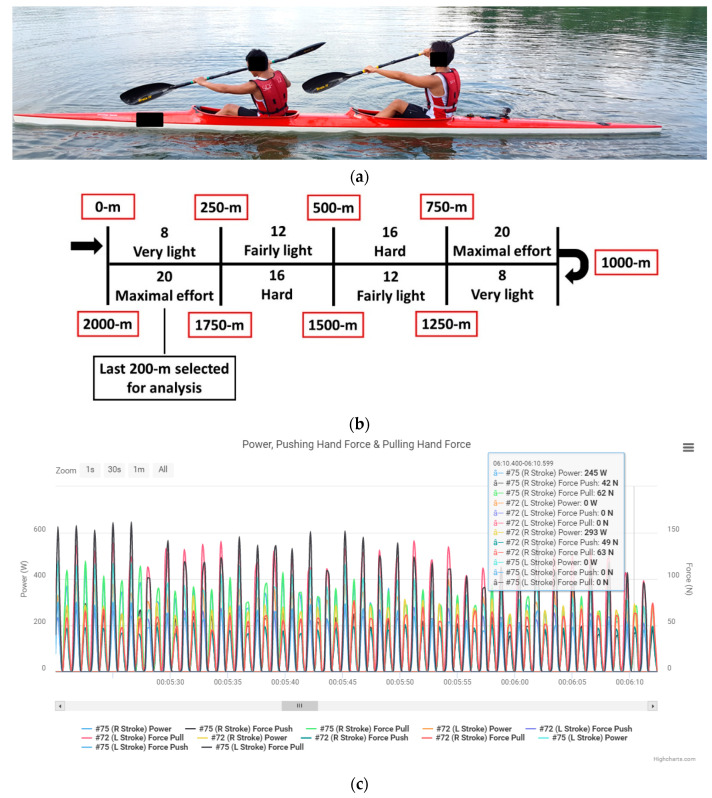
(**a**) K2 crew paddling on water with instrumented paddles; (**b**) step protocol with self-moderated intensities changing every 250-m; (**c**) raw data recorded using the analysis application (http://analysis.onegiantleap.co.nz).

Participants were asked to paddle two bouts of 2 km using self-paced intensities (Figure 2). The first bout was performed in K1, and the second in a K2 boat with their regular partner and in their preferred seat order (Figure 2a). This sequence replicated that a sprint kayaker would warm up in K1 before practicing crew boats. The experiment protocol was to change kayaking intensities every 250-m based on the 6-20 Borg scale [14]. The steps were 8 “very light”, 12 “fairly light”, 16 “hard”, and 20 “maximal effort”, and then the sequence was repeated (Figure 2b). Force and power data during the last 200-m of ‘maximal effort’ paddling were recorded using the analysis application (Figure 2c, http://analysis.onegiantleap.co.nz). Prior to each bout, participants were briefed again on the step protocol and encouraged to do their best. Between bouts, they had 20-min of passive recovery which included the time to adjust their seat and foot-rest fittings as needed. Participants used their own K1 and K2 boats that met the minimum competition weights of 12 kg and 18 kg, respectively. The total duration of the experiment for each crew was about 1 h.

### 2.4. Data Processing

#### 2.4.1. Filtering raw data

**Figure 3:**
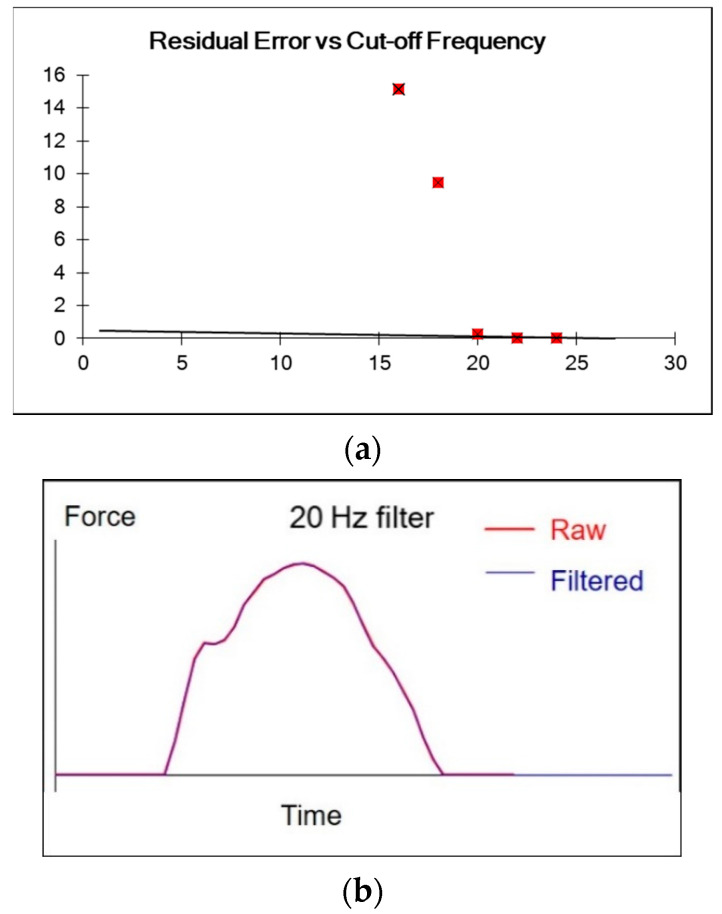
Data processing with a 4th order low-pass Butterworth filter: (**a**). Comparing among cut-off frequencies of 16, 18, 20, 22, and 24 Hz. (**b**). The 20 Hz filter was deemed to be the most suitable.

Stroke force and power data were transmitted and downloaded wirelessly in dat file format via the Windows Desktop App (Version 1.1.9.10). To extract the data, each file was uploaded sequentially onto a web-based application (http://analysis.onegiantleap.co.nz). In the literature, stroke force data have been processed with a 4th order low-pass Butterworth 20 Hz filter to smooth and remove random errors [3]. In the present study, visual inspection and residual analysis were performed to compare among cut-off frequencies of 16, 18, 20, 22, and 24 Hz (Figure 3). Subsequently, the value of 20 Hz was selected to filter all raw force and power data.

#### 2.4.2. Extraction of Key Stroke Variables

**Figure 4:**
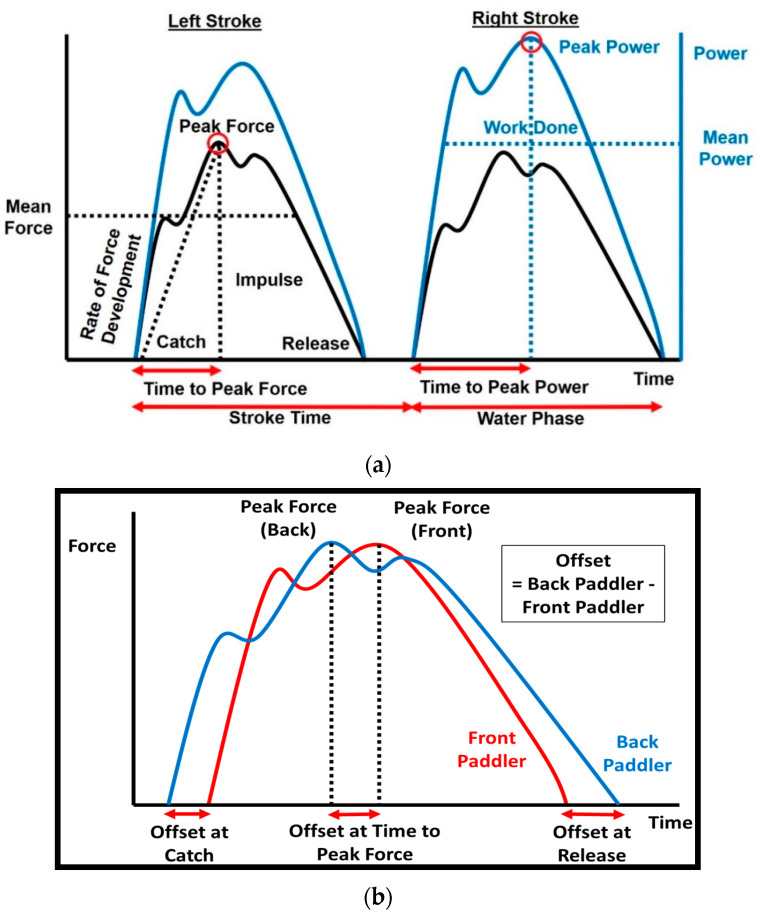
Extraction of key variables of interest for each stroke: (**a**). force, power, and temporal variables for each individual paddler; (**b**) timing offset variables between the front and back paddlers.

After filtering the raw data, key stroke variables of all strokes within the last 200-m maximal effort paddling were extracted using customised codes written in RStudio (version 1.0.136, R Core Team 2016, R Foundation for Statistical Computing, Vienna, Austria). Among the 74 participants, an average of 77 (SD = 5) strokes were analysed. The variables can be classified into force, power, and temporal variables (Figure 4), with reference from previous studies and recommendations [1,2,11,15,16]. For each stroke, the following force, power, and temporal variables were extracted:

- Force variables
  The force variables extracted were peak force, mean force, force ratio, rate of force development, impulse, and impulse rate (Figure 4a). Peak force is the maximum force within a stroke. Mean force is calculated over the water phase, and the aerial phase was excluded. Force ratio is the percentage of mean force to peak force. Rate of force development is peak force divided by the time to peak force. Impulse is the area under the force–time curve as derived from the trapezoid integration rule. Impulse rate is the product of impulse and stroke rate divided by 60 s. Impulse rate has been proposed by Baker [16] as an important stroke parameter for sprint kayaking because it better quantifies the change of momentum over a number of stroke cycles, as stroke impulse and stroke rate were thought to be inversely related.
- Power variables
  The power variables extracted were peak power, mean power, power ratio, and work done (Figure 4a). Peak power is the maximum power within a stroke. Mean power is calculated over the water phase and excluded the aerial phase. Power ratio is the percentage of mean power to peak power. Work done is the area under the power–time curve as derived from the trapezoid integration rule.
- Temporal variables
  For each individual kayaker, the temporal variables extracted were stroke rate, stroke time, water phase duration, time to peak force, and time to peak power (Figure 4a). Stroke time and water phase duration were defined according to the two-phase observational model by McDonnell et al. [17]. Stroke time was the duration from the catch of one stroke to the catch of the next stroke. Stroke rate was calculated by dividing 60 s by stroke time to obtain the number of strokes per min. To provide insights for the coordination strategies between the two crew members, timing offset variables were obtained at four instances of the stroke cycle: at the catch, time to peak force, time to peak power, and release (Figure 4b). An offset was defined as the timing difference of the back paddler with reference to the front paddler [12,13,18]. A zero offset indicates that the two paddlers are in perfect synchronisation.

### 2.5. Statistical Analyses

Statistical analyses were performed in IBM SPSS Statistics for Windows (version 26, IBM Corp, Armonk, USA). Descriptive results were presented as mean and 95% confidence intervals. For each stroke variable, an average value of all analysed strokes (mean = 77, SD = 5) within each participant was used for statistical analysis. A series of 2 × 3 two-way factorial Analysis of Variance was performed to examine the effects of seat (front vs. back) and group (national, recreational, school) on the key stroke variables. Effect size was calculated by partial eta squared, where η^2^ was interpreted as small (0.01), medium (0.06), and large (0.14) according to guidelines [19,20]. Where necessary, post hoc pairwise comparisons were performed and adjusted with Bonferroni’s correction. To examine the coordination between the front and back paddler, a one-way ANOVA was conducted to compare the effect of the group on the timing offset variables. All statistical tests were set at the 0.05 level.

## 3. Results

The mean boat speeds of the last 200-m “maximal effort” paddling showed significant differences among the three groups (*p* < 0.001), with the national team (4.5 (4.2, 4.8) m/s) faster than both recreational clubs (3.9 (3.8, 4.1) m/s) and school teams (3.7 (3.6, 3.8) m/s).

### 3.1. Kinetic Profiles

**Figure 5:**
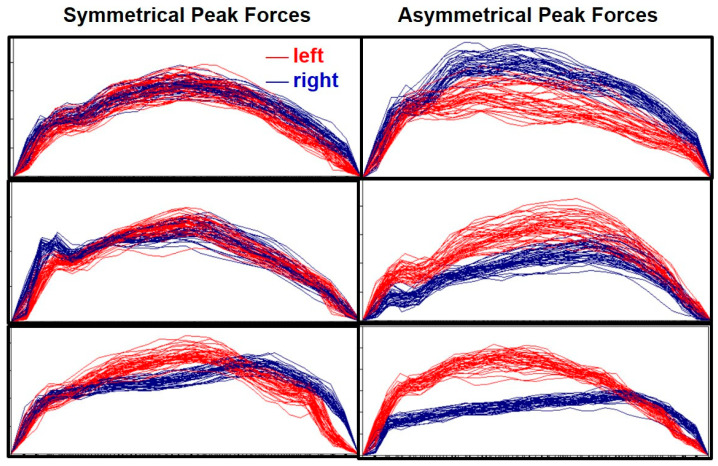
Examples of time-normalised stroke force profiles of the left (red) and right (blue) strokes. Each panel presents data of all analysed strokes within one participant during 200-m maximal effort paddling.

Among the 74 kayakers, different force profiles were observed (Figure 5). Within an individual, there existed various degrees of differences between the left and right sides, such as the magnitude of peak forces, timing of the peak force, and shape of the force–time curve.

### 3.2. Seat Position

There was no significant difference between the paddlers sitting in the front and back seats in force (Table 2), power (Table 3), or temporal (Table 4) variables.

### 3.3. Ability Level

Among the three groups of varied kayaking abilities, there were significant differences in most force (Table 2), power (Table 3), and temporal (Table 4) variables. For force variables, the national team kayakers displayed higher peak force, mean force, rate of force development, impulse, and impulse rate than the recreational and school athletes (Table 2). There was, however, no difference in force variables between recreational and school groups. Three out of four power variables differed between the national team and the recreational group as well as between the recreational and the school groups (Table 3). In terms of temporal variables, the national team athletes were characterised by higher stroke rate, and shorter stroke time and water phase duration when compared with the other two groups (Table 4). These national team athletes also showed shorter time to peak power than the school team athletes.

### 3.4. Coordination Strategy

The coordination between the front and back paddlers, as reflected by timing offset variables, did not differ among the national, recreational, and school groups (Table 5).

## 4. Discussion

The present study used instrumented paddles, the Kayak Power Meter, to conduct on-water investigation into sprinting kayaking performance in 74 kayakers paired in K2 crew boats. The main findings were: (1) there was no difference in force, power, or temporal characteristics between the paddlers sitting in the front and back seats, (2) proficient kayakers produced greater kinetic outputs than less proficient kayakers, and (3) the coordination strategy between the front and back crew members in a K2 boat was similar across ability levels.

### 4.1. Seat Position

This study showed that the kinetic outputs in a K2 crew boat were unaffected by the seat position, as no differences in force, power, or temporal variables were found between the front and back paddlers. This finding is in line with the earlier work by Gomes [11] which reported similar force profiles when the same kayaker sat in the front and back seats of a K2 crew boat. The study by Gomes [11], however, only investigated a small sample of 11 elite kayakers and did not compare the stroke kinetics between the K2 pairs when paddling with their usual partner in their preferred seat order. Our study on a larger cohort of 74 male kayakers provided empirical evidence that the front and back paddlers of K2 crews who regularly trained together exhibit similar stroke characteristics as their partner. Furthermore, this pattern of similar kinetic outputs is consistent across different ability levels from school athletes to competitive national team kayakers.

In another type of crew boat, the dragon boat, the front positions of the paddlers have been linked to increased injury risk [21]. Across 302 elite Iranian dragon boaters, the overall incidence was 10.2 injuries/1000 training sessions. By seat position, the paddlers at the front three rows of the boat accounted for a disproportional ~50% of injuries, while those at the last three rows made up only 20% of injuries. One reason suggested was that paddlers at the front experienced increased loads from catching into stagnant or highly turbulent water. Conversely, the back paddlers were catching into relatively steady backward flow caused by the motion of the boat and blades of the front paddlers. In the study by [21], however, no force data were collected to confirm the speculation that front paddlers experienced higher loads than the back paddlers. Based on findings from the present study, the front paddler in a K2 kayak did not experience greater load than the back paddler and therefore the front paddler should not have an increased risk of musculoskeletal injuries.

### 4.2. Ability Level

Clear differences in force, power, and temporal variables were seen among the three groups of kayakers of varied abilities. As expected, proficient kayakers were characterised by greater kinetic outputs than their less proficient counterparts, including higher peak force, mean force, rate of force development, impulse, impulse rate, peak power, mean power, and work done (Table 2 and Table 3). While most force variables differed between national team from the other two less proficient groups, these force variables cannot further differentiate the subtle difference between the recreational and school athletes. Interestingly, three out of four power variables were significantly higher in the recreational group than the school group. These results suggest that power variables are more sensitive than force variables to detect less obvious differences in kinetic outputs between kayakers of varied abilities.

In terms of temporal variables, the national team athletes were characterised by higher stroke rate, and shorter stroke time and water phase duration when compared with the other two groups (Table 4). These national team athletes also took a shorter time to reach peak power than the school athletes. In an observational study by Robinson et al. [22] on three K4 crews, stroke rate was highest in the fastest crew. The present finding of higher stroke rate in the national team compared with the less proficient kayakers suggest that stroke rate is important for good K2 performance. From our data, the fastest crew (crew 39, mean boat velocity of 4.8 m/s) comprised two members (participant 73 and 75) who paddled with the highest stroke rates (117.4 spm and 115.1 spm, respectively) among all participants. These results echo the findings from a recent study on individual K1 kayaking which showed that higher stroke rate was positively correlated with increased kayak velocity (*r* = 0.904, *p* < 0.001) [23]. The temporal results in K2 mirror some previous findings on individual K1 events [24,25,26]. For instance, high stroke rates have been strongly linked to success in K1 200-m [26,27]. In a review by McDonnell and colleagues [25], stroke time had the strongest correlation to 200-m race performance time (r = −0.86, *p* < 0.05) and water phase duration was highly associated with stroke time (r = −0.83, *p* < 0.05). Based on the current study and the literature, it appears that for both K1 and K2 sprint kayaking events, high stroke rate and short stroke time are common indicators for good performance.

Wearable sensors are becoming more and more important in sports to provide key data relating to training and competitive performance [28]. This study demonstrated that instrumented paddles can be applied to obtain useful performance feedback during on-water maximal effort paddling. These stroke-by-stroke kinetic data can be useful for coaches, sport scientists, and athletes in planning and monitoring the training. Based on the kinetic outputs, specific feedback and interventions can be targeted to improve a kayaker’s performance. The force–time profiles may also be useful in team selection, for example, for the pairing of K2 crew members with similar stroke characteristics.

### 4.3. Coordination Strategy

This study showed that the coordination strategy based on timing differences between the front and back paddlers was similar among the national, recreational, and the school groups (Table 5). The offset timings were generally small (31 ms to 68 ms, Table 5) which were comparable to the offset values of 34 ms at the catch position previously reported by Gomes [11] using a different type of instrumented paddle system sampled at 256 Hz. The lack of differences among the three groups of kayakers at varied ability levels suggest that timing offsets are not important indicators for K2 performances. This finding parallels other studies on K2 crews using video analysis that timing offsets between the front and back paddlers were largely unaffected by seat order [13] or visual obstruction [18]. Translating the research findings into practice, coaches are advised not to pay high attention to the timing difference between crew members in a K2 boat. Instead, emphasis should be placed on increasing kinetic outputs such as force and power. We acknowledge that the present study only examined the timing difference between the two paddlers at key events (i.e., catch, time to peak force, time to peak power, release) and this method may be over-simplistic to comprehensively evaluate the coordination strategy between crew members. Future study may consider performing more complex analysis such as examining the entire waveforms of the force- and power-time histories to explore the time-lag characteristics at a more in-depth level.

## 5. Conclusions

This study demonstrated that instrumented paddles can be useful tools to obtain performance feedback during on-water kayaking. Stroke-by-stroke kinetic data of each crew member in a K2 boat, as well as the coordination strategy between the crew members, can be closely monitored. The findings showed that the force, power, and temporal characteristics of the front and back paddlers were similar during maximal effort sprint kayaking. Proficient kayakers could produce greater kinetic outputs than less proficient kayakers, while the coordination strategy between the two crew members in a K2 boat was similar across ability levels. For practical advice, coaches need not pay high attention to timing difference between crew members in a K2 boat. Emphasis should be placed on increasing kinetic outputs such as force and power both crew members in a K2.

## Figures and Tables

**Table 1:** Physical characteristics and experience of 74 male K2 sprint kayaking athletes from the national team, recreational clubs, and school teams.

	National (N, *n* = 9)	Recreational (R, *n* = 38)	School (S, *n* = 27)	*p*	*Post-hoc*	
Age [years]	23.2 [20.1, 26.2]	24.0 [22.9, 25.2]	17.9 [15.7, 20.0]	<0.001 *	N > S	R > S
Height [m]	1.74 [1.71, 1.78]	1.73 [1.71, 1.74]	1.70 [1.68, 1.72]	0.033 *		
Body mass [kg]	73.4 [71.2, 75.5]	68.3 [65.9, 70.8]	61.8 [59.7, 64.0]	<0.001 *	N > S	R > S
Sitting height [m]	0.91 [0.88, 0.93]	0.91 [0.89, 0.92]	0.87 [0.86, 0.88]	0.001 *	N > S	R > S
Sitting reach [m]	1.26 [1.22, 1.29]	1.25 [1.23, 1.27]	1.22 [1.20, 1.24]	0.061		
Arm span [m]	1.77 [1.72., 1.82]	1.76 [1.74, 1.78]	1.73 [1.71, 1.76]	0.167		
Experience [years]	10.0 [6.5, 13.5]	5.7 [4.4, 7.0]	2.5 [1.8, 3.2]	<0.001 *	N > S	R > S

N = national team, R = recreational club. S = school team. Data are presented as mean [95% CI]. Significant difference (*p* < 0.05) is shown in bold text and indicated by an asterisk.

**Table 2:** Force variables of front and back paddlers of K2 crews during a 200-m maximal effort sprint kayaking.

				ANOVA						*Post-hoc*	
				Seat		Level		Interaction			
		Front	Back	*p*	*η* ^2^ *_p_*	*p*	*η* ^2^ *_p_*	*p*	*η* ^2^ *_p_*		
Peak force [N]	N	344.3 [307.6, 381.0]	342.4 [252.9, 432.0]	0.884	<0.001	<0.001 *	0.340	0.978	0.001	N > R	N > S
	R	239.6 [207.0, 272.1]	241.7 [219.6, 263,9]								
	S	216.9 [188.7, 245.1]	223.3 [182.6, 263.9]								
Mean force [N]	N	205.3 [187.2, 223.4]	200.6 [149.9, 251.4]	0.794	0.001	<0.001 *	0.329	0.974	0.001	N > R	N > S
	R	146.5 [130.0, 163.0]	146.6 [134.9, 158.3]								
	S	137.4 [120.0, 154.9]	135.3 [111.1, 159.6]								
Force ratio [%]	N	60.1 [57.1, 63.1]	58.9 [56.0, 61.8]	0.106	0.036	0.059	0.075	0.684	0.010	--	
	R	62.1 [60.1, 64.1]	61.2 [59.4, 63.1]								
	S	63.7 [62.1, 65.3]	61.3 [59.2, 63.4]								
RTD [N/s]	N	1929.8 [1460.3, 2399.2]	1699.3 [1197.3, 2201.2]	0.939	<0.001	<0.001 *	0.328	0.399	0.025	N > R	N > S
	R	1118.2 [938.6, 1297.8]	1233.0 [1016.1, 1449.9]								
	S	972.9 [831.1, 1114.7]	1065.6 [834.8, 1296.4]								
Impulse [Ns]	N	91.1 [84.5, 97.7]	88.1 [68.3, 107.8]	0.668	0.003	0.001 *	0.189	0.981	0.001	N > R	N > S
	R	71.1 [64.8, 78.6]	70.6 [64.5, 76.7]								
	S	68.8 [59.1, 78.6]	67.8 [56.4, 79.2]								
Impulse rate [Ns/s]	N	155.9 [133.4, 178.4]	150.9 [109.7, 192.0]	0.708	0.002	<0.001 *	0.372	0.967	0.001	N > R	N > S
	R	106.2 [94.1, 118.3]	104.3 [94.4, 114.1]								
	S	97.1 [84.9, 109.2]	96.6 [78.3, 115.0]								

N = national team, R = recreational clubs, S = school teams, RTD = rate of force development. Data are presented as mean [95% CI] Significant difference (*p* < 0.05) is shown in bold text and indicated by an asterisk.

**Table 3:** Power variables of front and back paddlers of K2 crews boat during a 200-m maximal effort sprint kayaking.

				ANOVA						*Post-hoc*		
				Seat Order		Level		Interaction				
		Front	Back	*p*	*η* ^2^ *_p_*	*p*	*η* ^2^ *_p_*	*p*	*η* ^2^ *_p_*			
Peak power [W]	N	924.8 [820.4, 1029.3]	970.3 [727.8, 1212.8]	0.525	0.006	<0.001 *	0.493	0.784	0.007	N > R	N > S	R > S
	R	627.9 [527.9, 728.0]	619.6 [547.1, 692.0]									
	S	480.3 [429.6, 530.9]	518.8 [458.9, 578.8]									
Mean power [W]	N	497.5 [440.6, 554.4]	516.4 [393.1, 639.7]	0.641	0.003	<0.001 *	0.513	0.924	0.002	N > R	N > S	R > S
	R	342.8 [300.9, 384.8]	342.7 [306.2, 379.2]									
	S	280.5 [250.0, 311.0]	288.2 [257.5, 319.0]									
Power ratio [%]	N	54.1 [51.7, 56.5]	53.7 [50.6, 56.7]	0.335	0.013	0.037 *	0.088	0.611	0.014	N < S		
	R	56.2 [53.7, 58.7]	55.9 [54.3, 57.5]									
	S	58.7 [57.2, 60.2]	56.4 [53.4, 59.4]									
Work done [J]	N	220.2 [199.1, 241.4]	226.4 [182.2, 270.6]	0.733	0.002	<0.001 *	0.499	0.794	0.006	N > R	N > S	R > S
	R	166.1 [150.7, 181.4]	162.6 [149.9, 175.3]									
	S	139.3 [125.2, 153.3]	143.9 [130.4, 157.4]									

N = national team, R = recreational clubs, S = school teams. Data are presented as mean [95% CI]. Significant difference (*p* < 0.05) is shown in bold text and indicated by an asterisk.

**Table 4:** Temporal variables of front and back paddlers of K2 crews during a 200-m maximal effort sprint kayaking.

				ANOVA						*Post-hoc*	
				Seat Order		Level		Interaction			
		Front	Back	*p*	*η* ^2^ *_p_*	*p*	*η* ^2^ *_p_*	*p*	*η* ^2^ *_p_*		
Stroke rate [spm]	N	102.6 [91.1, 114.0]	101.7 [91.0, 112.4]	0.904	<0.001	<0.001 *	0.336	0.986	<0.001	N > R	N > S
	R	88.6 [85.3, 91.9]	88.6 [85.3, 92.0]								
	S	85.3 [80.2, 90.4]	85.3 [80.3, 90.3]								
Stroke time [ms]	N	598.3 [533.8, 662.8]	596.7 [529.7, 663.6]	0.977	<0.001	<0.001 *	0.263	0.998	<0.001	N < R	N < S
	R	683.7 [657.8, 709.6]	683.2 [657.5, 708.8]								
	S	711.4 [665.0, 757.9]	712.1 [665.8, 758.5]								
Water phase duration [ms]	N	423.3 [391.7, 454.9]	425.0 [395.5, 454.5]	0.800	0.001	<0.001 *	0.202	0.826	0.005	N < R	N < S
	R	473.7 [453.9, 493.4]	463.7 [443.0, 484.4]								
	S	481.4 [457.6, 505.3]	482.1 [461.2, 503.0]								
Time to peak force [ms]	N	183.3 [155.4, 211.2]	205.0 [183.2, 226.8]	0.914	<0.001	0.061	0.075	0.230	0.040		
	R	221.1 [203.5, 238.6]	205.3 [188.5, 222.0]								
	S	225.7 [210.7, 240.7]	217.1 [196.2, 238.1]								
Time to peak power [ms]	N	173.3 [143.9, 202.8]	195.0 [177.8, 212.2]	0.874	<0.001	0.038 *	0.087	0.143	0.053	N < S	
	R	202.6 [191.1, 214.2]	190.5 [179.1, 201.9]								
	S	214.3 [196.6, 231.9]	201.4 [181.6, 221.3]								

N = national team, R = recreational clubs, S = school teams, spm = strokes per minute. Data are presented as mean [95% CI]. Significant difference (*p* < 0.05) is shown in bold text and indicated by an asterisk.

**Table 5:** Timing offset variables between the front and back paddlers of K2 crews during a 200-m maximal effort sprint kayaking.

	National (N, *n* = 6)	Recreational (R, *n* = 19)	School (S, *n* = 14)	*p*	*Post-hoc*
Catch [ms]	41.7 [26.2, 57.1]	38.9 [29.6, 48.3]	31.4 [25.9, 36.9]	0.196	--
Time to peak force [ms]	45.0 [25.4, 64.6]	58.9 [48.2, 69.7]	67.9 [55.2, 80.5]	0.108	--
Time to peak power [ms]	36.7 [17.1, 56.2]	44.7 [36.6, 52.8]	52.9 [43.7, 62.0]	0.131	--
Release [ms]	38.3 [14.0, 62.6]	39.5 [29.1, 49.8]	38.6 [29.8, 47.3]	0.988	--

Data are presented as mean [95% CI].
